# Dominant egg surface bacteria of *Holotrichia oblita* (Coleoptera: Scarabaeidae) inhibit the multiplication of *Bacillus thuringiensis* and *Beauveria bassiana*

**DOI:** 10.1038/s41598-021-89009-6

**Published:** 2021-05-04

**Authors:** Kui Wang, Qi Liu, Chunqin Liu, Lili Geng, Guirong Wang, Jie Zhang, Changlong Shu

**Affiliations:** 1 Guangdong Laboratory for Lingnan Modern Agriculture (Shenzhen Branch), Genome Analysis Laboratory of the Ministry of Agriculture, Agricultural Genomics Institute at Shenzhen, Chinese Academy of Agricultural Sciences, Shenzhen, 518000 China; 2 State Key Laboratory for Biology of Plant Diseases and Insect Pests, Institute of Plant Protection, Chinese Academy of Agricultural Sciences, Beijing, 100193 China; 3 Cangzhou Academy of Agricultural and Forestry Sciences, Cangzhou, 061001 China

**Keywords:** Pathogens, Microbiome

## Abstract

*Holotrichia oblita* (Coleoptera: Scarabaeidae) and some other scarab beetles are the main soil-dwelling pests in China. *Bacillus thuringiensis* (Bt) and *Beauveria bassiana* (Bb) are entomopathogens that have been used as biocontrol agents of various pests. However, scarab larvae especially *H. oblita* exhibited strong adaptability to these pathogens. Compared to other scarabs, *H. oblita* could form a specific soil egg case (SEC) structure surrounding its eggs, and young larvae complete the initial development process inside this structure. In this study, we investigated the role of SEC structure and microorganisms from SEC and egg surface in pathogen adaptability. 16S rRNA gene analysis revealed low bacterial richness and high community unevenness in egg surface, with *Proteobacteria*, *Firmicutes*, *Bacteroidetes* and *Fusobacteria* dominating. In terms of OTUs composition analysis, the data show that the egg surface contains a large number of unique bacteria, indicating that the egg bacterial community may be derived from maternal transmission. Furthermore, we found that all culturable bacteria isolated from egg surface possessed antimicrobial activity against both Bt and Bb. The *Pseudomonas* bacteria with a significantly higher abundance in egg surface showed strong Bt- and Bb antagonistic ability. In conclusion, this study demonstrated a unique and antimicrobial bacterial community of *H. oblita* egg surface, which may contribute to its adaptability. Furthermore, the specific SEC structure surrounding the *H. oblita* eggs will provide a stable microenvironment for the eggs and egg surface bacteria, which probably provides more advantages for *H. oblita* adaptation ability.

## Introduction

*Holotrichia oblita* (Coleoptera: Scarabaeidae) and some other scarab beetles are the main soil-dwelling pests in China, which cause significant economic losses in agriculture, horticulture, and forestry. The larvae living in soil, known as white grubs, feed on the underground parts of most crops in the field such as sweet potatoes, soybeans, peanuts. And adults damage the leaves of trees and field crops^1^. Chemical pesticides are often used to control grubs, but the extensive use of chemicals leads to serious soil pollution and poses a threat to the human and ecological health^2^. Soil not only supports plant and animal life, but also hosts myriad microorganisms inside, including many entomopathogenic microorganisms, some of which have been isolated and applied as biocontrol agents, such as *Beauveria bassiana* (Bb) and *Bacillus thuringiensis* (Bt)^3^. To date, many Bt and Bb isolates have been reported to have activity against scarab beetles^4–7^.

In the past ten years, our institute has done a lot of works on biological properties and efficient control strategies of these scarab pests, and previous data indicate that scarab larvae exhibit strong adaptability to pathogens^6,7^. The field grub population always causes significant losses, although there are a plenty of entomopathogenic microbes in soil. And when applying Bt and Bb agents in the field, the scarab pests especially *H. oblita* usually need more dosage than leaf-feeding Lepidoptera pests^6–9^.

Bb and Bt have different modes of action. The Bb species attack their host insects generally percutaneously, which can directly penetrate through the insect cuticle by germination of the spores and proliferation within the host by formation of hyphal bodies/blastospores^10^. Different from Bb, Bt functions as a stomach insecticide. The main Bt virulence factors are the parasporal crystals proteins (Cry) produced during sporulation^11^. In addition, Bt can produce other virulence factors during vegetative growth stage, such as vegetative insecticidal proteins (Vip), bacteriocins, chitinases and enhancins^8,12^. Therefore, Bt cell also contributes to its insecticidal activity. Garbutt et al. also confirmed that Bt cell with stronger proliferation ability in the insect body would significantly enhance the insecticidal activity^13^. Additionally, Bt cell can proliferate in the rhizosphere of plants where the scarabs lay their eggs, which means that more insecticidal proteins and virulence factors are produced and exposed to the eggs and larvae^14^. Therefore, we hypothesize that the scarab adaptability against pathogens might be correlated with their ability of inhibiting the multiplication of Bt and Bb.

**Figure 1:**
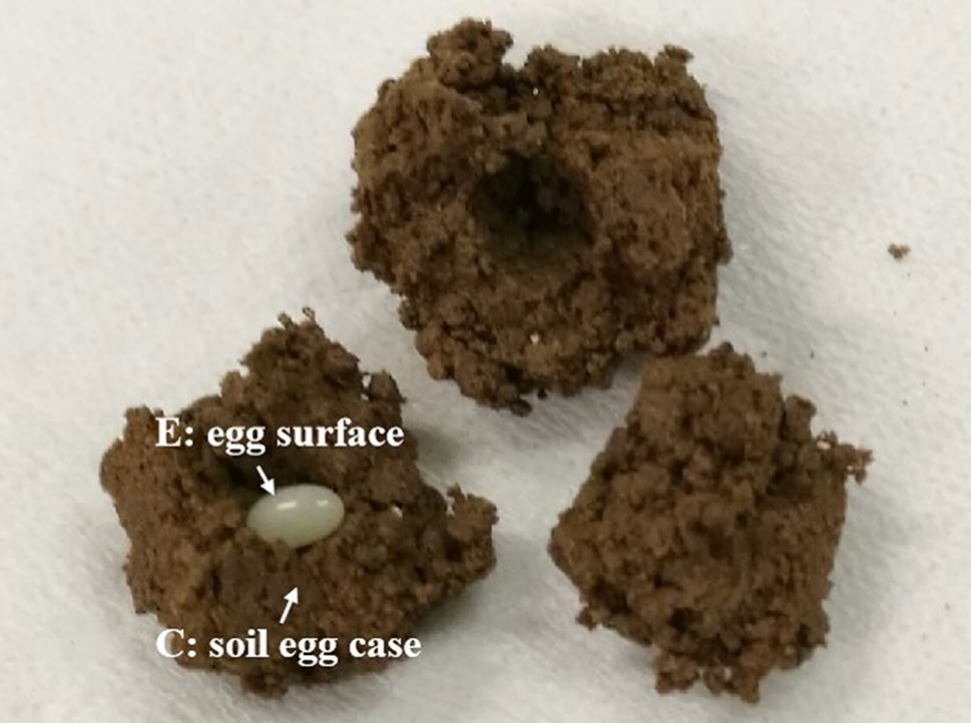
Photograph of the SEC structure and egg of *H. oblita*. SEC (C) samples and egg surface (E) samples were collected as shown in the Figure, and bulk soil (B) samples were collected from the soil about 10 cm away from the egg surface.

Young larval stages are more sensitive to the pathogen^7,15^. Under natural conditions, scarab adults usually lay their eggs in the soil near their preferred host plants, where the newly hatched larvae are exposed to the rich soil microflora including these entomopathogenic microorganisms. Therefore, we investigate the biological features of scarab eggs and larvae. And we find that *H. oblita* egg is coated with a unique soil egg case (SEC) structure, which is different from two other scarab pests *H. parallela* and *Anomala corpulenta* (see Fig. 1). Previous data indicated that invertebrate eggs were usually defensed by the protective systems provided by maternal organisms. For example, tick eggs were protected by the antimicrobial factors on the egg surface^16,17^; spider eggs were protected well by the silk cocoon provided by the female spiders^18^. Therefore, in this work, to understand the role of SEC structure and egg bacteria in pathogen adaptability, we analyzed the microbial community structure of *H. oblita* egg surface and SEC structure respectively using 16S rRNA gene sequencing; furthermore, we tested the antimicrobial potential of the cultivable isolates from egg surface and SEC against scarab-specific Bt and Bb strains. The results suggest that egg surface has a unique bacterial and antimicrobial community, contributing to the low susceptibility of young larvae against entomopathogenic pathogens. And the specific SEC structure provides protection for the eggs and hatchlings of *H. oblita*, which is beneficial for better adaptation ability of *H. oblita*.

## Results

### Microbial diversity comparisons between egg and soil samples

**Figure 2:**
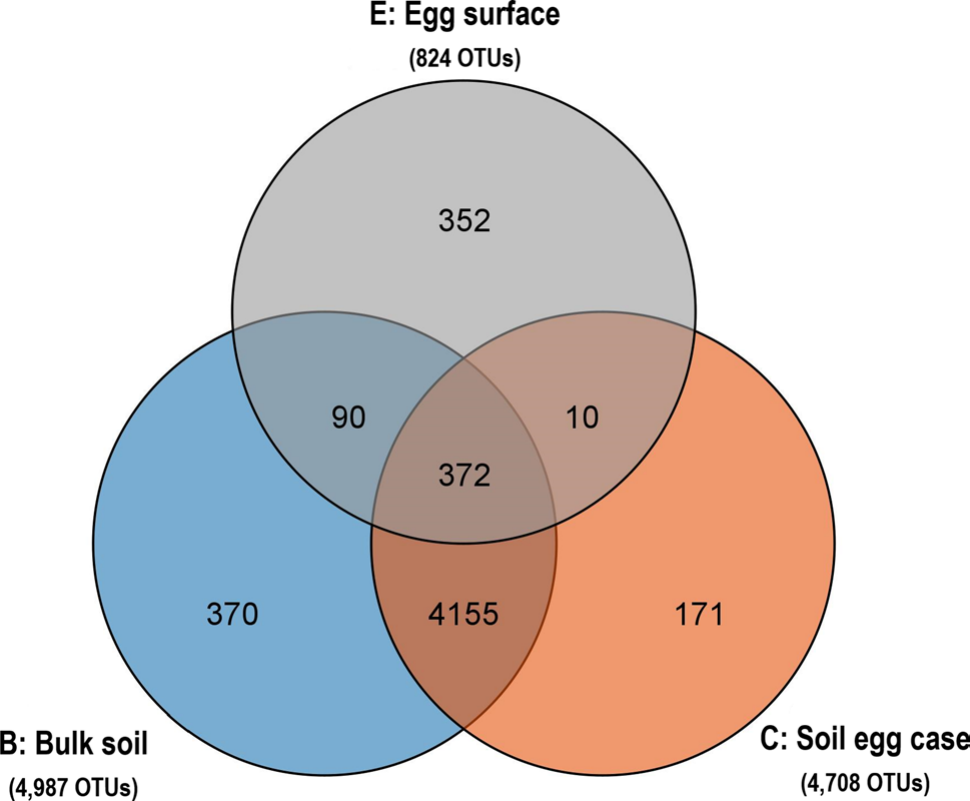
Comparison of OTUs numbers among different samples.

The bacterial composition of different samples from *H. oblita* egg surface (E), SEC (C), and bulk soil (B) (Fig. 1) was determined by sequencing analysis of the 16S rRNA gene. A total of 2,748,824 raw reads were generated from 20 samples, including 667,427 raw reads from six E samples, 924,724 raw reads from seven C samples, and 1,156,673 raw reads from seven B samples. After removing the short reads and trimming the low-quality regions, a total of 1,894,784 effective tags were identified, with an average length of 415 bp (Table S1). All the effective sequences were grouped at 97% DNA sequence similarity, and an average of 3545, 3261 and 311 OTUs were obtained from bulk soil (B), soil egg case (C) and egg surface (E) samples, respectively (Table 1). A total of 5520 non-redundant OTUs were identified from bulk soil (4987 OUTs), SEC structure (4708 OTUs) and egg surface (824 OTUs), respectively (Fig. 2). Among the 824 OTUs identified in egg surface, 352 unique OTUs (42.72%) were not detected in both bulk soil and SEC structure.

**Table 1:** Mean ± SD (standard error) of alpha diversity indexes of OTUs from different samples. Different letters in the same column indicate significant differences among samples at *p* = 0.05.

Sample	Shannon	OTUs number	Chao1	Simpson	Dominance	Equitability
Bulk soil (B)	9.18 ± 0.17^a^	3545 ± 124.14^a^	3545.84 ± 124.06^a^	0.008 ± 0.001^b^	0.992 ± 0.001^a^	0.778 ± 0.011^a^
Soil egg case (C)	9.24 ± 0.16^a^	3261 ± 310.52^a^	3262.34 ± 310.32^a^	0.008 ± 0.002^b^	0.992 ± 0.001^a^	0.792 ± 0.011^a^
Egg surface (E)	4.54 ± 0.40^b^	311 ± 46.97^b^	311.90 ± 47.24^b^	0.105 ± 0.024^a^	0.895 ± 0.024^b^	0.549 ± 0.040^b^

Alpha diversity analysis was then performed to assess the diversity and evenness of the microbial population from different samples. The alpha diversity patterns were variable across the bulk soil (B), SEC (C) and egg surface (E) samples (Table 1). The number of observed OTUs and alpha diversity analysis based on Shannon and Chao1 indexes in egg surface (E) significantly decreased than SEC (C) and bulk soil (B), indicating that soil samples had more microbial diversity than the egg surface samples (Table 1). A previous study in our laboratory showed that the microorganism collection method could affect the community structure, where the phyllosphere community diversity was lower for samples subjected to DNA extraction than for those subjected to direct PCR^19^. In the present study, we performed direct PCR for egg surface (E) samples and added a DNA extraction process before PCR for SEC (C) and bulk soil (B) samples. The results confirmed that the community diversity of soil samples was much higher than the egg surface samples. The results of Simpson, Dominance and Equitability indexes indicated that, compared to SEC (C) and bulk soil (B) samples, the evenness of egg surface (E) decreased (Table 1). The rarefaction curve based on the Shannon index showed that all samples reached a plateau, suggesting that our sampling effort was sufficient to obtain a full estimate of OTU richness (Figure S1).

**Figure 3:**
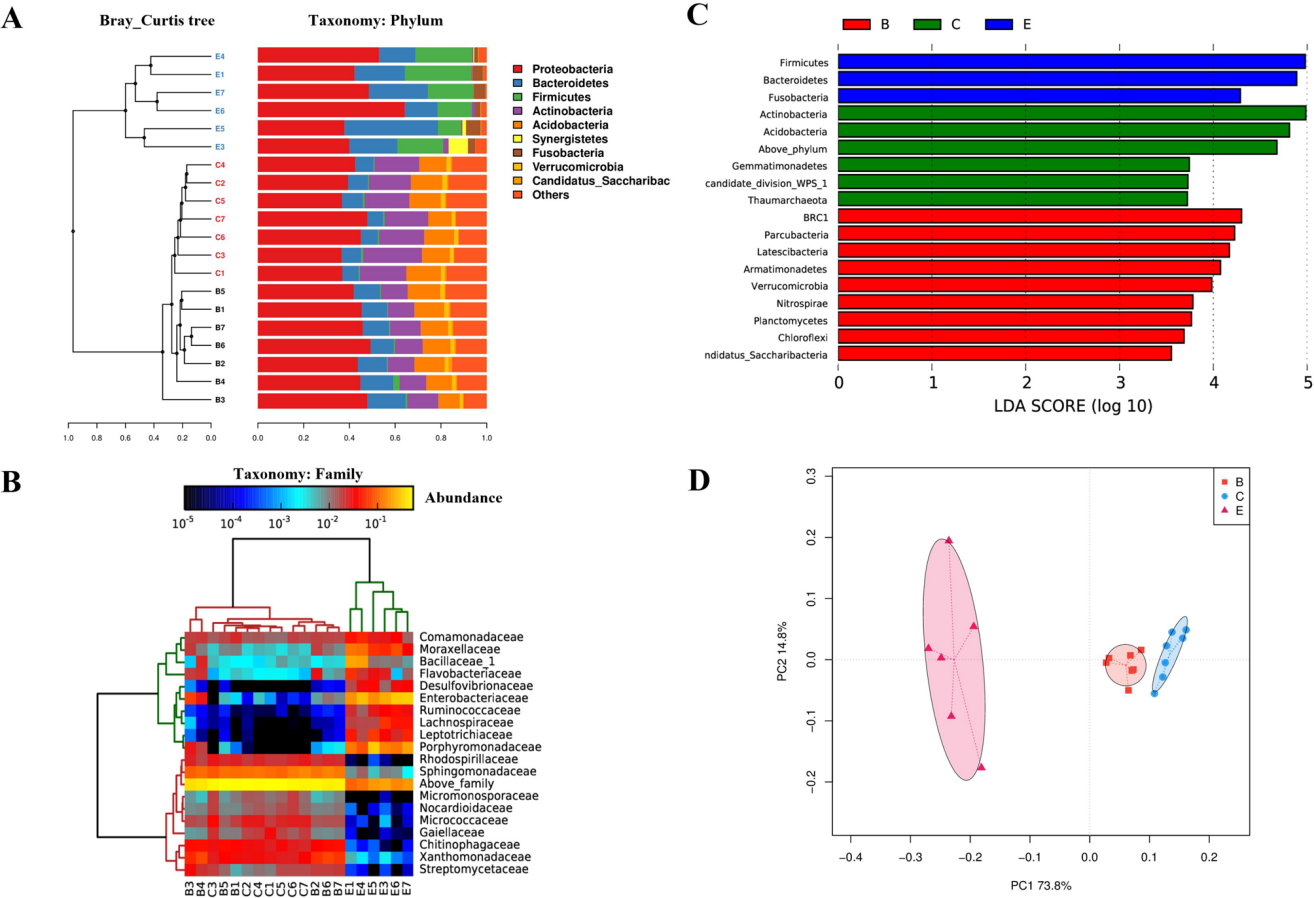
Taxonomic composition of microbial communities in different samples (B: bulk soil, C: SEC and E: egg surface). (**A**) Relative abundance of the microbes at phylum level and (**B**) family level respectively. The sample variation in the community structure is also highlighted in the Bray–Curtis tree (**A**) and cluster tree (**B**). (**C**) Comparison of microbiota using Lefse (LDA) test at the phylum level. LDA scores represent the significant microbial difference among different samples. (**D**) PCA analysis for microbiota variability at the phylum level. Each symbol represents a sample. The variance explained by the PCs is indicated on the axes.

Among all samples, 26 phyla, 143 families, and 300 genera were identified. *Proteobacteria* was the dominant phylum and comprised most of all detected microorganisms (approximately 44.63%) (Fig. 3A). This is typically observed in other soil libraries^20,21^. *Actinobacteria*, *Acidobacteria* and *Bacteroidetes* were also abundant in SEC (C) samples and bulk soil (B) samples. In egg surface (E) samples, *Firmicutes*, *Bacteroidetes*, and *Fusobacteria* were the most abundant phyla (Fig. 3A). The community structure varied markedly among different samples, outlined by the Lefse LDA results (Fig. 3C). Compared with bulk soil (B) samples, the composition of *Firmicutes*, *Bacteroidetes*, *Fusobacteria* and the composition of *Actinobacteria*, *Acidobacteria* significantly increased in egg surface (E) samples and SEC (C) samples, respectively (Fig. 3C). Bray–Curtis tree and PCA analysis also indicated that microbiota in different samples were clearly separated at the phylum level (Fig. 3A,D). PC1 and PC2 explained 73.8% and 14.8% of the global variation, respectively (Fig. 3D). Similar results were observed in the NMDS analysis based on Weighted UniFrac distances (Figure S2).

At the family level, Sphingomonadaceae and Xanthomonadaceae in phylum *Proteobacteria* and Chitinophagaceae in phylum *Bacteroidetes* were enriched in bulk soil (B) samples. Rhodospirillaceae in phylum *Proteobacteria* and Micrococcaceae in phylum *Actinobacteria* were enriched in SEC (C) samples. The families Enterobacteriaceae, Moraxellaceae, and Desulfovibrionaceae in phylum *Proteobacteria*, Porphyromonadaceae in phylum *Bacteroidetes*, Leptotrichiaceae in phylum *Fusobacteria*, Ruminococcaceae and Lachnospiraceae in phylum *Firmicutes* were enriched in egg surface (E) samples (Fig. 3B). Differences were also observed at the class, order and genus level (Figure S3).

**Figure 4:**
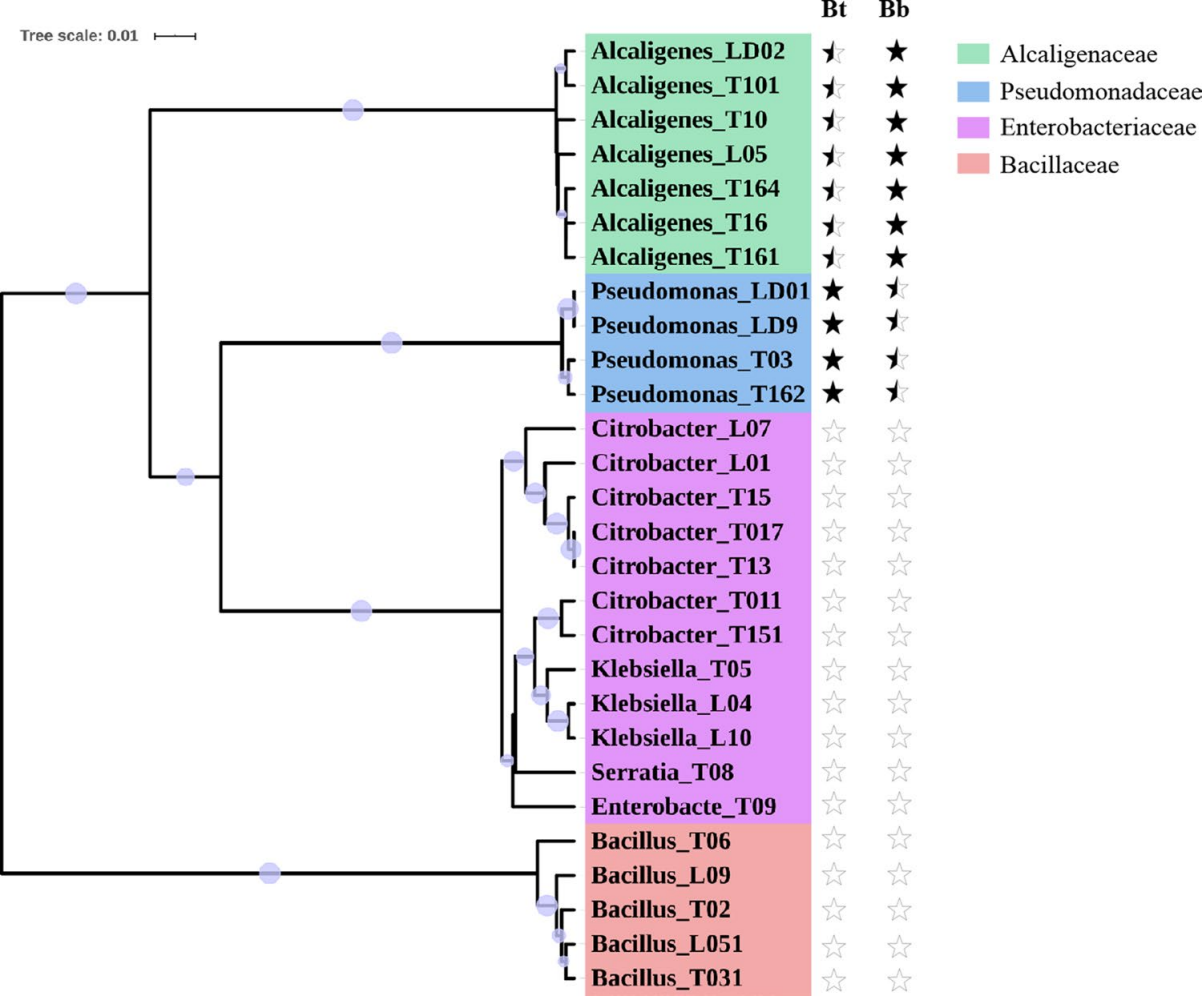
Phylogenetic analysis of 28 cultivable isolates. The tree is constructed based on the 16S rRNA gene sequences of each strain. Bootstrap values over 0.5 (1000 replications) are indicated using filled purple circles on the branch. Strain ID prefixed with “LD” indicates egg surface (E) isolates, strain ID prefixed with “L” indicates SEC (C) isolates, and strain ID prefixed with “T” indicates bulk soil (B) isolates. Different label background colors represent different clades at the family level. The antimicrobial activity against “Bt” or “Bb” strains is indicated using pentagram symbols, filled black pentagrams represent strong antimicrobial activity, partially filled pentagrams represent weak antimicrobial activity, and open pentagrams represent no antimicrobial activity.

From 20 samples, we isolated 28 strains with different colony morphology and found the number of cultivable isolates from bulk soil samples (18 strains from B) was much higher than SEC samples (7 strains from C) and egg surface samples (3 strains from E). Then we performed 16S rRNA gene sequencing to identify these 28 isolated strains. All the sequences were aligned against the NCBI database using BLAST, and the results showed that these 28 isolates belonged to two phyla, *Proteobacteria* and *Firmicutes*. Phylogenetic analysis based on the 16S rRNA sequences revealed that these 28 isolates clustered into four major groups at the family level, i.e., Alcaligenaceae, Pseudomonadaceae, Enterobacteriaceae, and Bacillaceae (Fig. 4). Alcaligenaceae, Enterobacteriaceae, and Pseudomonadaceae belonged to the *Proteobacteria* phylum, which constituted the largest group (23 isolates). The other five Bacillaceae strains belonged to the *Firmicutes* phylum (Table S2).

The 18 isolates from bulk soil (B) samples were composed of 7 different genera, *Alcaligenes*, *Citrobacter*, *Bacillus*, *Pseudomonas*, *Klebsiella*, *Enterobacter*, and *Serratia*. The seven isolates from SEC (C) samples were composed of four genera, *Alcaligenes*, *Bacillus*, *Citrobacter*, and *Klebsiella*. The three isolates from egg surface (E) samples were composed of two genera, *Alcaligenes* and *Pseudomonas* (Table S2).

### The effects of cultivable isolates against pathogens

**Figure 5:**
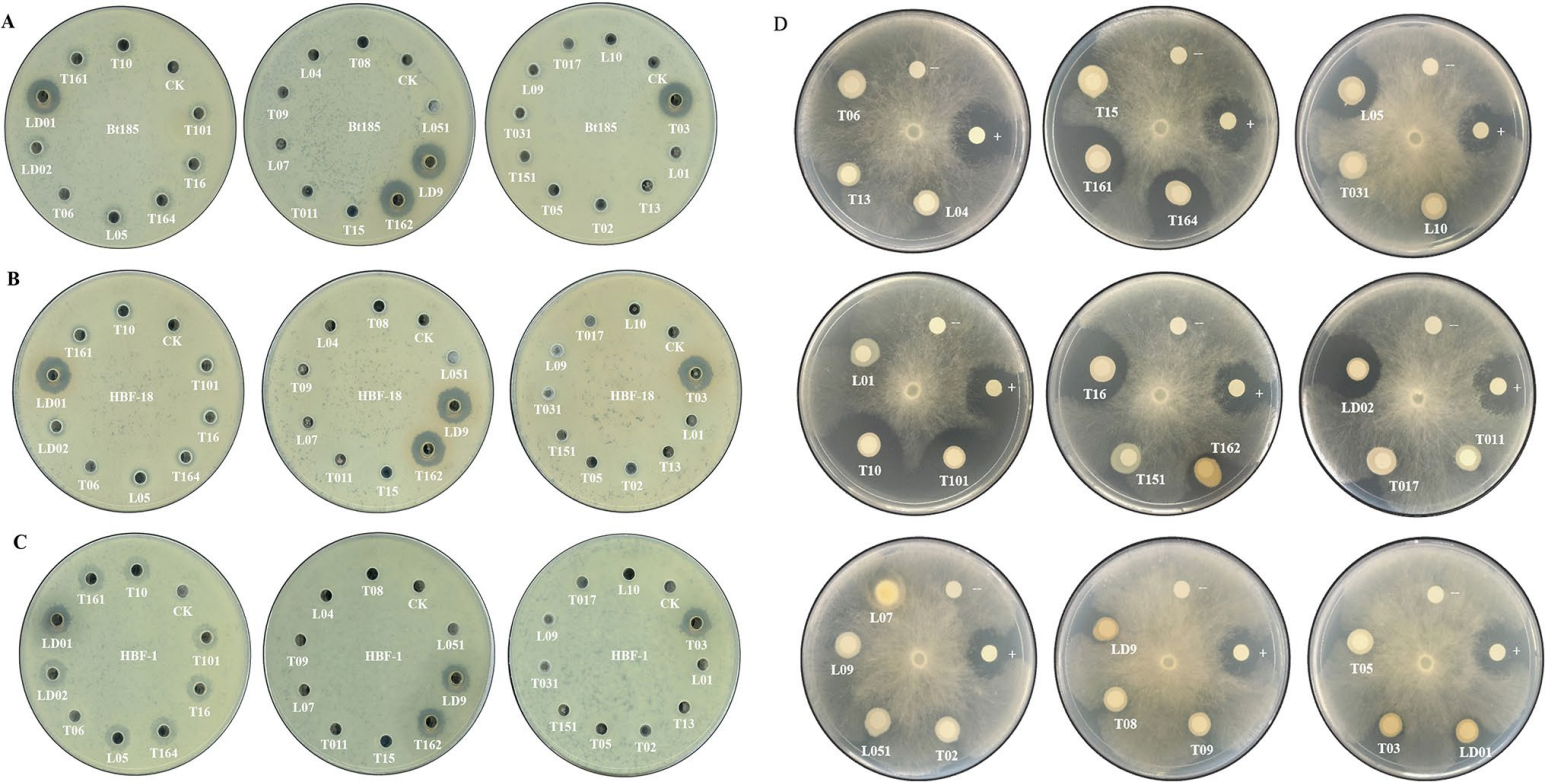
Effect of 28 cultivable isolates against the scarab-specific Bt strain Bt185 (**A**), HBF-18 (**B**), HBF-1 (**C**), and Bb strain (**D**). CK in (**A**), (**B**), and (**C**) and symbol “−” in (**D**) signifies negative control. The symbol “+” in (**D**) signifies positive control. Strain ID prefixed with “LD” indicates egg surface isolates, strain ID prefixed with “L” indicates SEC isolates, and strain ID prefixed with “T” indicates bulk soil isolates.

We assessed the antimicrobial activity of the 28 cultivable isolates against scarab-specific Bt and Bb strains. The confrontation culture analysis showed that strains (LD01, LD9) from *H. oblita* egg surface (E) samples and strains (T03, T162) from bulk soil (B) samples had strong antagonistic ability against all three scarab-specific Bt strains and weak antagonistic ability against the Bb strain. All these four strains were *Pseudomonas*. Strain LD02 from E samples, strain L05 from C samples, and strains (T10, T16, T101, T161, T164) from B samples showed weak antagonistic ability against all three Bt strains but showed strong antagonistic ability against the Bb strain. These seven strains belonged to *Alcaligenes*. The remaining 17 strains showed no antagonistic ability against the Bt and Bb strains, including 12 *Proteobacteria* strains and 5 *Firmicutes* strains (Fig. 5 and Table S2).

All the three isolates from E samples showed antagonistic ability (100%, N = 3) against pathogens, where the proportions of antimicrobial isolates in B and C samples were 38.89% (N = 18) and 14.29% (N = 7), respectively.

### Genome sequencing and secondary metabolite analysis of strains with antimicrobial activity

**Figure 6:**
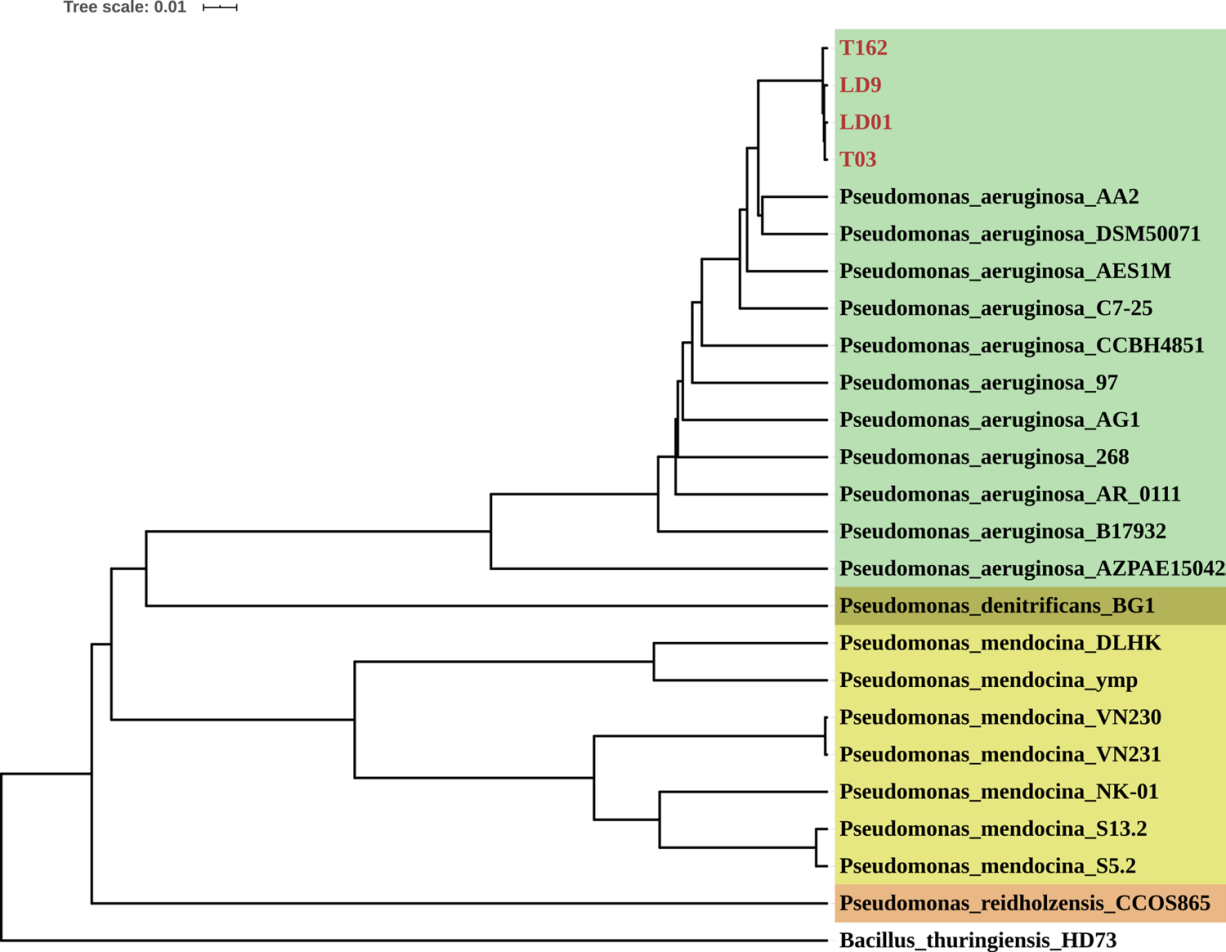
Phylogenetic analysis of four *Pseudomonas* strains (LD01, LD9, T03, and T162). This tree is constructed based on the whole genome of each strain. Different label background colors represent different clades at the species level. Bt strain Bacillus_thuringiensis_HD73 is used as the outgroup.

The four strains (LD01, LD9, T03, and T162) with strong Bt-antagonistic ability and weak Bb-antagonistic ability were genome sequenced using the Illumina platform. After assembly and gene predication, 5885, 5857, 5850 and 5859 protein-coding sequences (CDS) were identified from LD01, LD9, T03 and T162, respectively (Table S3). The 16S rRNA gene sequence identification showed that these four strains belonged to the genus *Pseudomonas* and had the highest similarity with *P. aeruginosa* strain DSM50071 (99.51–99.79%). Therefore, we collected 20 additional *Pseudomonas* strain genomes from the NCBI GeneBank database (http://www.ncbi.nlm.nih.gov/), including 11 *P. aeruginosa* strains, 7 *P. mendocina* strains, 1 *P. denitrificans* strain, and 1 *P. reidholzensis* strain (Fig. 6 and Table S4). The whole-genome-based phylogenetic tree was constructed using CVTree and PHYLIP, with Bt *kurstaki* strain HD73 as an outgroup. The CVTree is an alignment-free method where each organism is represented by a Composition Vector (CV) derived from all proteins present in its genome. CVTree has been effectively used in several phylogenetic studies of microorganisms including archaea, prokaryotes, and fungi^22–24^. The results showed that these four strains were clustered with *P. aeruginosa* strains, indicating they belonged to *P. aeruginosa*. The blue-green coloration produced during culture verified this result. Phylogenetic analysis also showed high genome similarity among these four *P. aeruginosa* strains, suggesting that they might be the same strain. As an opportunistic human pathogen, *P. aeruginosa* can be isolated from various sources, including humans, animals, hospitals, swimming pools, soil, rhizosphere, and plants^25^. *P. aeruginosa* is also a promising biocontrol agent for plant pathogens and pests such as *Pythium* sp. and the root-knot nematode (*Meloidogyne incognita*)^26,27^. Nga et al. found that *P. aeruginosa* isolated from the rhizosphere of a watermelon plant showed high antagonistic ability against both bacterial and fungal pathogens on rice, watermelon, and cabbage^28^. Our study showed that *P. aeruginosa* also had antagonistic ability against entomopathogenic Bt and Bb strains.

Then we used antiSMASH 2.0 pipeline to identify and annotate the putative secondary metabolite biosynthesis gene clusters in the four strains. A total of 62 gene clusters were identified, including 18 NRPS (non-ribosomal peptide synthetase cluster), 9 NRPS-like fragments, 8 hserlactone (homoserine lactone cluster), 7 bacteriocin, 8 phenazine, 4 CDPS (tRNA-dependent cyclodipeptide synthases), 4 NAGGN (N-acetyl-glutaminyl-glutamine-amide), and 4 thiopeptides (Table S5).

## Discussion

In China, although scarab pests cause significant yield reductions and economic losses each year, but due to the difficulty of these insect rearing in laboratory conditions, researches on these scarab beetles are very limited. Our institute has been focused on the biological properties and efficient control strategies of scarab pests for many years, and previous data indicate that scarab larvae exhibit strong adaptability to pathogens. Smith’s work on fossil record has demonstrated that Scarabaeoidea is quite resilient to external environment. Scarab beetles belong to polyphagan, the group of which first appears in the Triassic and has a family-level extinction rate of zero for most of their evolutionary history^29^. These data suggest that the scarab insects have a strong environment adaptability, which can be a challenge to the control of this group pests.

The environment adaptability was divergence among different scarab pests^6–8,30,31^. Both *H. oblita* and *H. parallela* belong to *Melolonthinae*, with similar morphological features and ecological taxonomic status, but the different adaptation abilities against pathogens indicate that they might have different environment adaptation strategy. Through biological characteristic analysis of these two scarabs, we find that *H. oblita* can form a specific SEC structure surrounding the eggs which *H. parallela* cannot. And the young *H. oblita* larva completes the initial development process inside this structure, which provides a relatively stable microenvironment beneficial to the development of the eggs and the stability of egg surface bacteria community. In this paper, beta diversity analysis indicated that the community of SEC structure is affected by the eggs and its surface bacteria. Therefore, according to the antimicrobial activity of egg surface bacteria, we speculate that this SEC structure possesses less anti-pathogen bacteria and provides protection for the eggs and hatchlings of *H. oblita*.

Female insects can vertically transmit to their offspring many beneficial bacteria which help the young hatchling inhibiting microbial competitors and pathogens, through different mechanisms. For example, the *Plataspidae* females (Heteroptera) enable their hatchlings acquire their gut symbiont by depositing symbiont capsules on the underside of the egg mass^32^; dung beetles transmit the symbionts to their larvae vertically by maternal fecal secretions deposited in the dung balls together with eggs^33^. In the present work, 16S rRNA sequencing analysis indicated that *H. oblita* egg surface exhibited a unique microbial community feature with significantly lower microbial diversity and significantly higher community unevenness. Furthermore, 42.72% OTUs (N = 872) in egg surface cannot be detected in both bulk soil and SEC structure, indicating that the unique bacterial community might originate from maternal transmission. Community composition analysis indicated that these bacteria possessed potential antimicrobial activity against pathogens. At the genus level, the composition of *Clostridium*, *Enterococcus*, *Pseudomonas*, *Acinetobacter*, *Desulfovibrio*, *Delftia*, *Sphingobium*, *Brevundimonas*, *Comamonas*, *Dysgonomonas*, *Emticicia*, *Empedobacter* and *Sebaldella* significantly increased in egg surface (Table S6), some of which have also been reported to exhibit antimicrobial activity against various pathogens. For example, many species of genus *Pseudomonas* have been proved synthesize a variety of compounds with antagonistic activity^28,34^; species of *Delftia* and *Sphingobium* produce antimicrobial compounds, which inhibit the growth of some common pathogenic microbes^35,36^. The further confrontation culture analysis confirmed the antimicrobial activity of egg surface bacteria against Bt and Bb, and the secondary metabolite analysis demonstrated the potential biosynthesis ability of antimicrobial compounds in these anti-pathogen isolates. For example, phenazines were reported to have antibiotic properties toward many bacteria and fungi and can damage mammalian cells^37,38^; thiopeptide antibiotics are a prominent class of antimicrobials with potent activity against gram-positive bacteria and many drug-resistant pathogens^39^. Therefore, these egg surface bacteria could reduce the pathogen infection probability through inhibiting the multiplication of Bb and Bt. Additionally, egg surface bacteria may help the larvae build a beneficial intestinal microbiota. Under natural conditions, the newly hatched larvae have a great chance to contact and ingest these microorganisms on the egg surface and the nearby soil, and some species that can colonize in the intestine will form the intestinal microbiota. The previous study has demonstrated that scarab larvae gut isolates exhibit antimicrobial activity against Bt strains, including these species with predominance in egg surface, such as *Acinetobacter*^40^.

Summarizing, the egg surface has a unique and antimicrobial bacterial community, which might originate from maternal transmission, contributing to the adaptability of scarabs. And the specific SEC structure surrounding the *H. oblita* eggs will provide a stable microenvironment for the eggs and egg surface bacteria, which probably provide a better adaptation ability for *H. oblita*.

## Methods

### Sampling and DNA extraction

*H. oblita* was collected from a field in Cangzhou, Hebei Province, China. The adults were reared in plastic boxes (66 cm by 41 cm by 18 cm) filled with soil containing willow leaves at a temperature of 25 ℃ until they laid eggs. SEC (C) samples and egg surface (E) samples were collected as shown in the Fig. 1, and bulk soil (B) samples were collected from the soil about 10 cm away from the egg surface.

SECs were collected and peeled off, then the eggs were transferred to a sterile 2 ml plastic centrifuge tube containing 1 ml sterile water and sonicated for 5 min in an Bransonic CPX Ultrasonic Cleaning Bath (BRANSON, USA) to dislodge bacteria. After centrifugation at 10,000×*g* for 5 min, the microorganisms in the wash buffer were collected and defined as the egg surface (E) sample, and resuspended with 1 ml sterile water. The SEC soils (1 g) and soils (1 g) 10 cm away from the SECs were suspended in 5 ml sterile water and centrifuged at 10,000×*g* for 5 min, and the pellets were defined as the SEC (C) sample and the bulk soil (B) sample and resuspended with 1 ml sterile water. A total of 20 samples were collected, including 6 E samples, 7 C samples, and 7 B samples.

For egg surface (E) samples, 1 μl of the microorganism suspension was directly used as a template for PCR. For soil samples (B and C), a 900 μl suspension was used to extract genomic DNA, using a PowerSoil DNA Isolation kit (MO BIO Laboratories, USA), and 1 μl of DNA was used as a template for PCR amplification. The remaining 100 μl suspension of 20 samples was kept for conventional culture using solid Luria Bertani (LB) agar medium, and single colonies were picked from the plates and repeatedly grown on solid agar plates until pure cultures were obtained. A total of 28 cultivable isolates were collected, including 3 isolates from E samples, 7 isolates from C samples, and 18 isolates from B samples. Genomic DNA of each isolate was extracted as previously described^41^.

### 16S rRNA gene sequencing and bioinformatic analysis

The V3–V4 region of microbial 16S rRNA genes of 20 samples were amplified by PCR using the specific primers, 341F (5′-CCTAYGGGRBGCASCAG-3′) and 806R (5′-GGACTACNNGGGTATCTAAT-3′). PCR products were purified using a QIAquick Gel Extraction Kit (QIAGEN, Germany). The TruSeq DNA PCR-Free Sample Preparation Kit (ILLUMINA, USA) was used for 16S rRNA gene amplicon library construction. The Qubit 2.0 Fluorometer (Thermo Fisher SCIENTIFIC, USA) and Agilent 2100 Bioanalyzer (Agilent Technologies, USA) were used for library quality assessment. Finally, the library was sequenced on the Illumina HiSeq 2500 sequencer (ILLUMINA, USA), and 250 bp paired-end reads were generated. Raw Data were trimmed using Trimmomatic (version 0.36)^42^ with default parameters. Then the clean paired-end reads were assembled into raw tags using Usearch (version 9.2.64)^43^. The primer sequences in the raw tags were trimmed, and effective tags were obtained. The 16S rRNA sequence data of 20 samples were deposited in Sequence Read Archive (SRA) database under BioProject ID PRJNA637400, with accession number SRR11931252–SRR11931271.

The operational taxonomic units (OTUs) were clustered at 97% identity cutoff with a Usearch UPARSE algorithm^44^. Then the chimera sequences were removed based on the UPARSE pipeline analysis. The OTU annotation was performed using the Usearch SINTAX algorithm^45^, against RDP training set (version 16) 16S rRNA Database with a confidence threshold of 0.8. OTUs annotated as chloroplast or mitochondria or OTUs not annotated to the kingdom level were abandoned. QIIME (version 1.7.0) pyNAST algorithm^46^ was used for species annotation against the GreenGene Database^47^. Usearch (version 9.2.64)^43^ was used to calculate Alpha diversity metrics, including the indexes (Shannon, Chao1) reflecting the sample community richness, and indexes (Simpson, Dominance, and Equitability) reflecting the sample community evenness. QIIME (version 1.7.0)^48^ was used to calculate beta diversity to estimate variation between samples. Principal Component Analysis (PCA) and Non-Metric Multi-Dimensional Scaling (NMDS) analysis were performed using R package (https://www.r-project.org/) to visualize complex relationships between samples. Lda Effective Size (LEfSe) test for variability of microbiota was calculated using lefse (Version 1.0.7) (http://huttenhower.sph.harvard.edu/galaxy/).

For 28 cultivable isolates, the 16S rRNA genes of each isolate were amplified using the specific primers, 27F (5′-AGAGTTTGATCMTGGCTCAG-3′) and 1492R (5′-TACCTTGTTACGACTT-3′). Then the 16S rRNA sequences were identified through aligning against NCBI 16S rRNA sequence (Bacteria and Archaea) database with BLAST (https://blast.ncbi.nlm.nih.gov/Blast.cgi).

### Confrontation culture analysis

Four entomopathogen strains were used: 3 scarab-specific Bt strains (HBF-1, HBF-18, Bt185) ^6,49,50^, and one Bb strain BBNS-J9-16 (preservation number: CGMCC No.5288). The dual culture tests for antagonistic ability of 28 cultivable isolates against Bt strains were processed using the cup-plate confrontation culture method, as previously described^40^. Sterile water was used as a negative control. The observable inhibition zones were used as indicators of the antibacterial activity of the 28 isolates against Bt strains.

For the antagonistic ability analysis of the 28 isolates against Bb strain, the isolates were cultured at 30 ℃ with shaking at 220 rpm. The Bb strain was cultured on PDA for 2–4 days. Subsequently, fungal culture plugs were placed in the middle of LB agar plates. Five dishes of sterile blotter paper (6 mm diam.) were placed on the surface of the plate and inoculated with 10 μl of the cultured bacterial suspension. The amphotericin-B and sterile water were used as positive and negative controls, respectively. The plates were incubated at 28 ℃ for 5 days, and inhibition zones induced by 28 isolates against Bb strain were recorded.

### Genome sequencing and secondary metabolite analysis

Four cultivable isolates with strong antagonistic ability against Bt strains and weak antagonistic ability against Bb strain were selected for draft genome sequencing, using the Illumina HiSeq 2500 sequencer (ILLUMINA, USA). The produced reads were cleaned by removing reads with Ns or more than 20% low-quality bases, and 1 Gb 2 × 100 bp pair-end clean reads for each isolate were obtained. The Megahit (v 1.2.9)^51^ was used for genome assembly with default parameter, and QUAST (v 5.0.2)^52^ was used for quality assessment for genome assembly. In addition, the Prodigal (v 2.6.3)^53^ was performed for gene prediction. And the antiSMASH 2.0 pipeline^54^ was used for secondary metabolite analysis of these four isolates. The genome sequence data of four isolates were deposited in NCBI database under BioProject ID PRJNA715633, with accession number JAGFLW000000000, JAGFLX000000000, JAGFLV000000000, and JAGFLU000000000.

### Phylogenetic analysis

All the 16S rRNA sequences of 28 cultivable isolates were analyzed using MEGA (version 7)^55^ and an online tool iTOL: Interactive Tree of Life (http://itol.embl.de/)^56^. The analysis included bootstrapping values with 1000 replications. For the phylogenetic analysis of four genome sequenced isolates, we constructed the whole-genome-based tree using CVTree^22^ with *k-string* = 6, and PHYLIP^57^. The iTOL^56^ was used to annotate the tree.

## Supplementary Information

Supplementary Figures.

Supplementary Tables.
