# Supplementary material for: Dominant egg surface bacteria of Holotrichia oblita (Coleoptera: Scarabaeidae) inhibit the multiplication of Bacillus thuringiensis and Beauveria bassiana
Source: Sci Rep. 2021 May 4;11:9499. doi: 10.1038/s41598-021-89009-6 (PMC8096819; doi:10.1038/s41598-021-89009-6)
Supplement: Supplementary file 1 — Supplementary Figures. [file 41598_2021_89009_MOESM1_ESM.doc]

Supplementary Information

**Dominant egg surface bacteria of *Holotrichia oblita* (Coleoptera: Scarabaeidae) inhibit the multiplication of *Bacillus thuringiensis* and *Beauveria bassiana***

Kui Wang ^1,2^, Qi Liu ^2^, Chunqin Liu ^3^, Lili Geng ^2^, Guirong Wang ^1,2^, Jie Zhang ^2^ & Changlong Shu ^2*^

^1^ Guangdong Laboratory for Lingnan Modern Agriculture (Shenzhen Branch), Genome Analysis Laboratory of the Ministry of Agriculture, Agricultural Genomics Institute at Shenzhen, Chinese Academy of Agricultural Sciences, Shenzhen 518000, China

^2^ State Key Laboratory for Biology of Plant Diseases and Insect Pests, Institute of Plant Protection, Chinese Academy of Agricultural Sciences, Beijing 100193, China

^3^ Cangzhou Academy of Agricultural and Forestry Sciences, Cangzhou 061001, China

^*^Correspondence**:** clshu@ippcaas.cn


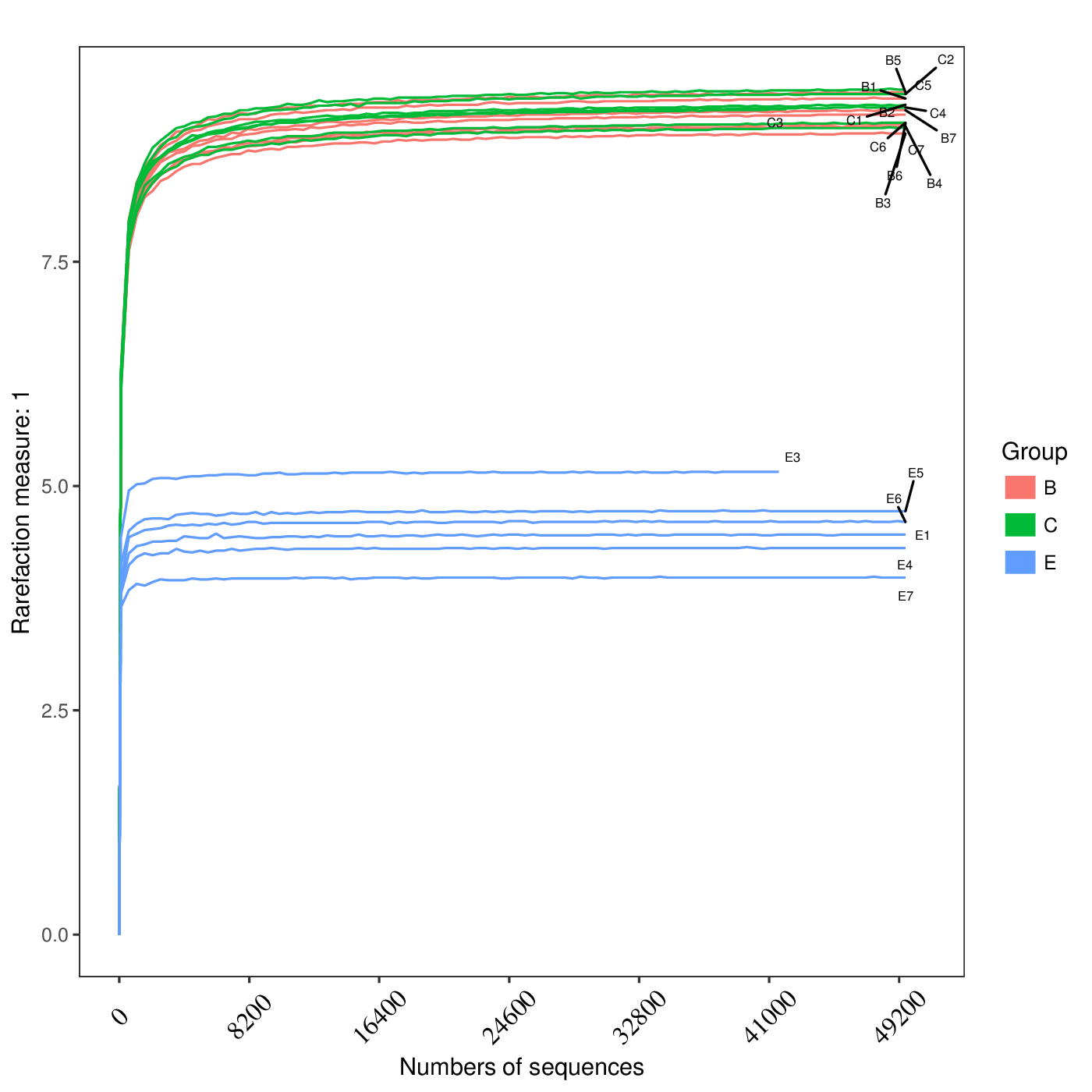


**Supplementary Figure S1.** Alpha diversity rarefaction curves based on Shannon index among 20 different samples (B: bulk soil, C: soil egg case, E: egg surface).


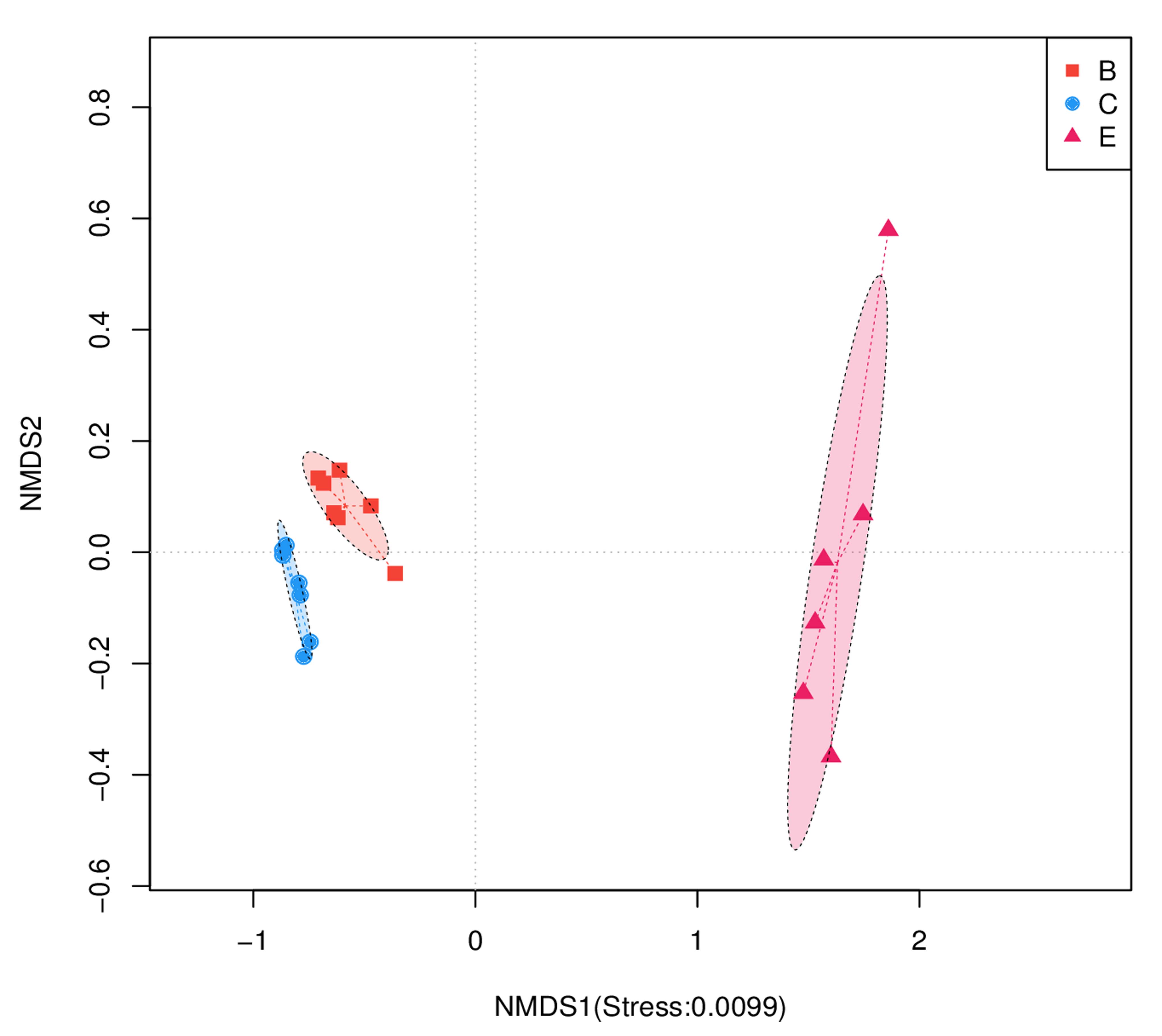


**Supplementary Figure S2.** NMDS analysis based on Weighted Unifrac distances among 20 different samples (B: bulk soil, C: soil egg case, E: egg surface). Each symbol represents a sample. The variance explained by the NMDSs is indicated on the axes.


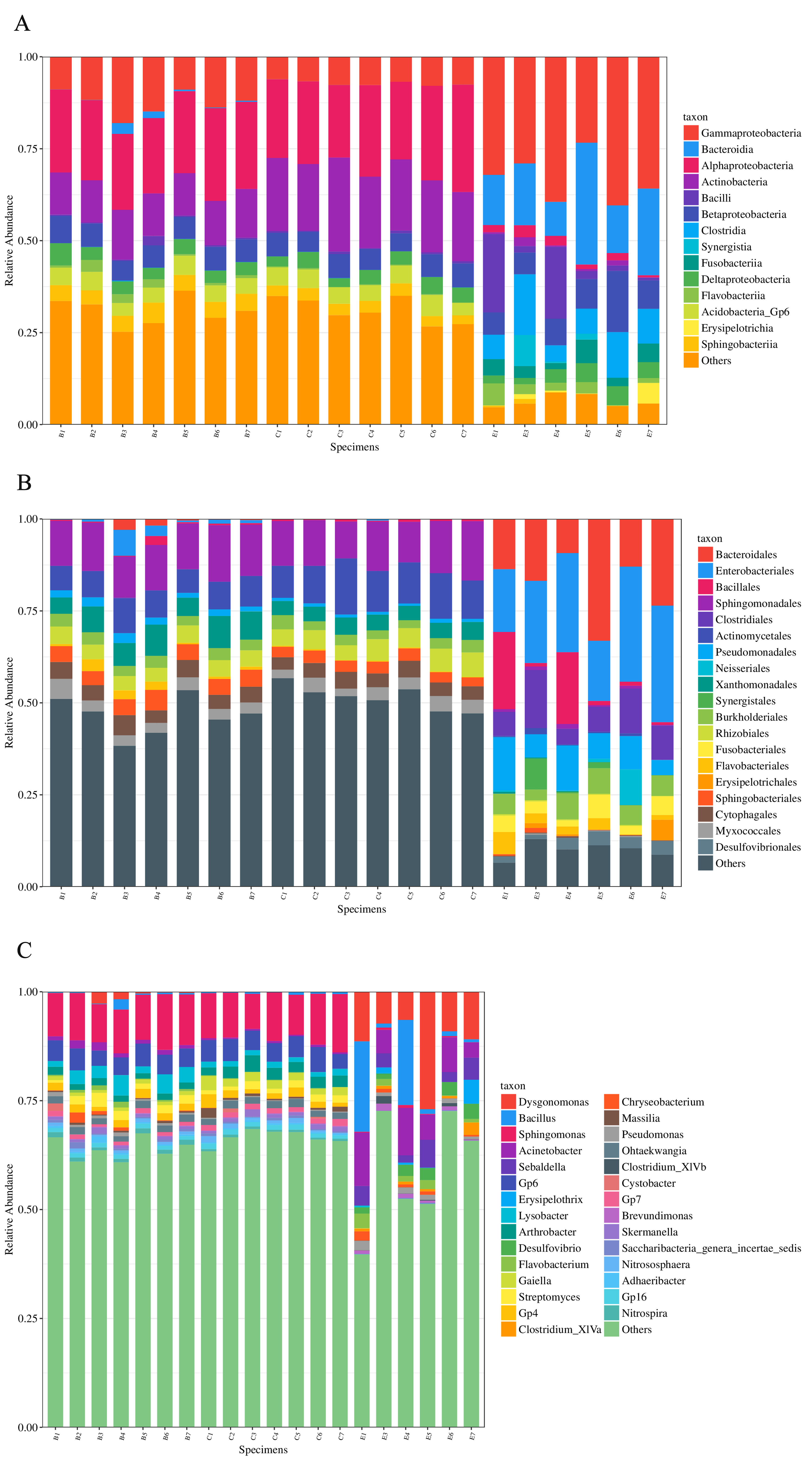


**Supplementary Figure S3.** Relative abundance of the microbes at class (**A**), order (**B**), and genus (**C**) level among 20 different samples (B: bulk soil, C: soil egg case, E: egg surface).
